# Mucins and Asthma: Are We Headed to the Revolutionary Road?

**DOI:** 10.3390/jcm8111955

**Published:** 2019-11-13

**Authors:** Pierachille Santus, Dejan Radovanovic, Davide Alberto Chiumello

**Affiliations:** 1 Department of Biomedical and Clinical Sciences (DIBIC), Division of Respiratory Diseases, Università degli Studi di Milano, Ospedale L. Sacco, ASST Fatebenfratelli-Sacco, Via G.B. Grassi, 74-20157 Milano, Italy; pierachille.santus@unimi.it (P.S.);; 2 SC Anestesia e Rianimazione, Ospedale San Paolo—Polo Universitario, ASST Santi Paolo e Carlo, Dipartimento di Scienze della Salute, Università degli Studi di Milano, Via Antonio di Rudinì, 8-20142 Milano, Italy; 3 Centro Ricerca Coordinata di Insufficienza Respiratoria, 20123 Milano, Italy

**Keywords:** asthma, MUC5AC, MUC5B, mucociliary clearance, T2 inflammation

## Abstract

Mucus represents the first line of defense of our respiratory tract and mucociliary clearance is essential for maintaining the homeostasis of airway epithelium. The latter mechanisms are altered in asthma and mucus plugging of proximal and distal airways is the main cause of death in cases of fatal asthma. Starting from the influential review performed by Luke R. Bonser and David J. Erle in 2017, we discuss the latest evidence in terms of mucins regulation and potential treatment of mucus hypersecretion and tissue remodeling in severe asthma.

The main cause of death in patients with fatal asthma is mucus plugging in proximal and distal airways [1,2]. This notion by itself should set mucus hypersecretion among the most relevant therapeutic targets in asthma. The patho-biology of mucus regulation, however, is a complex and intricated matter.

Considering an average breathing pattern, the air passing through the bronchi can easily exceed 10,000 L per day, exposing the respiratory tract to more than 20 million particles per hour, a lot more so in active smokers [3]. The first line of defense of our respiratory tract against this harmful storm of microbes, particulate matter, and dust is the production of mucus. In fact, the thin hydrogel that covers the airways’ surface acts as a physiological fly-paper that entraps airborne particles and irritants and maintains the homeostasis of the ciliated epithelium, avoiding dehydration and granting insulation, pH buffering, air humidification, and the adequate ecological niche for the airway microbiota [3,4]. Together with the periciliary layer, the mucus gel and the ciliated epithelium contribute to the continuous clearance of bacteria and viruses towards the upper airways, a process called mucociliary clearance. Mucociliary clearance is essential for maintaining an uninfected and unobstructed airway, but when it becomes dysfunctional, such as in asthma or chronic obstructive pulmonary disease (COPD), the hypersecretion and accumulation of mucus and the changes in its biophysical properties can become the major contributor to respiratory diseases.

Mucins are glycoproteins produced by goblet cells in the epithelium and by sero-mucous glands in the submucosa; mucins form the mucous hydrogel and are responsible for its functional properties. Of the numerous mucin (MUC) genes, only nine are expressed in the human respiratory tract, with MUC5AC and MUC5B having a key role in both health and disease [3]. The production of the mucins MUC5B and MUC5AC is mainly regulated by two genes adjacent to each other on chromosome 11p15.5 and is also controlled by the epidermal growth factor (EGF) signaling cascade. In patients with asthma, the abnormal expression of MUC5B, MUC5AC, and EGF receptor is correlated with disease severity. In asthma, mucus hypersecretion and accumulation represent the major determinant of airflow obstruction and airway hyper-responsiveness [5,6]. In fact, numerous mucin transcriptional alterations can impact airway surface pH, mucus adhesivity, and mucociliary transport [7]. Patients with type (T2)-high signature asthma consistently show an increased production of MUC5AC, a process mediated by both IL-13 and EGF. Moreover, epithelial remodeling in patients with mild to severe asthma is dominated by a dysregulated replication of goblet cells. Increases in goblet cell number accompany changes in mucin gene expression, which result in altered mucus composition and organization. These changes are associated with increased gel viscoelasticity and are sufficient to impair mucus transport through MUC5AC tethering, likely contributing to airway obstruction and mucus plugging [7].

The pathophysiological and clinical importance of the mucins MUC5AC and MUC5B in asthma have been brilliantly reviewed by Luke R. Bonser and David J. Erle in a seminal paper published in November 2017 in the *Journal of Clinical Medicine* [7]. Since then, the manuscript has received numerous citations for its clarity and the translational cut the authors gave to the review. The publication by Bonser and Erle, rather than being a simple overview of mucin role in asthma, left many open questions in terms of lacking therapeutic approaches, a challenge that has been taken on by numerous scientists and research groups worldwide.

In 2018, Shrine and colleagues [8] reported the results of the largest ever genome-wide association study of moderate-to-severe asthma. Genotyping patient-level data from two UK cohorts (the Genetics of Asthma Severity and Phenotypes [GASP] initiative and the Unbiased BIOmarkers in PREDiction of respiratory disease outcomes [U-BIOPRED] project), the authors found a shared genetic architecture between mild and moderate-to-severe asthma but also discovered three novel significant signals associated with the susceptibility to the development of moderate-to-severe asthma, specifically, in the MUC5AC region, in the transcription factor GATA3 (linked to the T-cell response in asthma and eosinophilia), and in the KIAA1109 locus. Finding variants in multiple genes related to T2 inflammation reinforces the rationale behind the targeting of pathways related to type 2 inflammatory processes. The molecular signaling that drives the expression of MUC5AC leads to the hypothesis that the novel and upcoming monoclonal antibodies for T2-high severe asthma, targeting IL-4 and IL-13 (namely, dupilumab) or regulating IL-13 production through thymic stromal lymphopoietin (TSLP), namely tezepelumab [9], may have a role in modulating mucus production in severe asthmatics. Prostaglandin D2 (PDG2) contributes to T2 inflammation through binding to the G-protein-coupled receptor chemoattractant receptor-homologous molecule expressed on TH2 cells (CRTH2). The activation of this pathway has potent downstream effects including mucus hypersecretion and airway remodeling [10,11]. Fevipiprant is an oral competitive antagonist of CRTH2 and, together with the GB001 compound [12], is the most promising oral drug currently under investigation for patients with moderate to severe asthma and a T2 inflammatory profile [10,12].

Upregulation of *MUC5AC* genes has been demonstrated also in animal models of T2-low (neutrophilic) and obesity-related asthma [13,14], thus shifting the possibility of a therapeutic approach also for patients without a T2 signature. In this view, a novel PDE4 (TAS-203) has shown favorable results in animal models of asthma, suppressing EGF-induced mucin MUC5AC expression and reducing goblet cell hyperplasia and MUC5AC production in the bronchoalveolar lavage fluid [15]. Tiotropium, currently approved for the add-on therapy in patients with moderate to severe asthma, has demonstrated some in vivo regulatory effects on mucus production [16], but, to date, any conclusion on the clinical significance of these effects seems premature. The upcoming phase III trials of triple inhaled therapy in patients with asthma may contribute to some extent to answer this question. Finally, severe asthmatics with mucus hypersecretion may benefit from non-pharmacological treatment approaches such as bronchial thermoplasty [17], but the evidence to date needs confirmation. Due to the lack of effective treatments, a remarkable amount of research has been lately employed to identify possible potent MUC5AC inhibitory agents among natural compounds, such as flavonoids, glycoside, and steroid-like molecules, that demonstrated modulatory effects on mucin expression, secretion, and production [18,19]. Some line of evidence previously suggested that *Pseudomonas aeruginosa*, a microorganism frequently responsible for acute and chronic lung infections in patients with COPD or bronchiectasis, activates mucus hypersecretion and MUC5AC expression through the EGF receptor pathway [20]. Recent advances in engineered glycoproteins may offer the chance to recreate synthetic mucins to study host–microbiome interactions and eventually employ mucin mimetics to prevent bacteria from forming biofilms or to domesticate virulent microbial populations bypassing the selective pressure that drives drug resistance [4].

Very recently, a group of researchers lead by David J. Erle demonstrated the possibility to selectively target SPDEF [21], a genetic domain which encodes a transcription factor previously shown to be essential for the differentiation of MUC5AC-producing goblet cells, by means of a single-guide RNA in human bronchial epithelial cells. The authors showed that the specific targeting of SPDEF abolished IL-13-induced MUC5AC expression and goblet cell differentiation, suggesting SPDEF as a potential target for mucus-regulatory therapies.

We believe that in the last years, research on mucin expression and regulation has received a great impulse and will certainly progress in the future. The rationale beyond mucin pharmacological targeting is strong, and there is a need for alternative efficacious treatments for severe asthma, especially for patients with T2-low inflammation. The effects of the available and upcoming compounds seem promising, but better patient selection, based on genomic profiling, would probably improve treatments’ efficacy and safety. In fact, we should not ignore that the depletion of goblet cells by dupilumab is also the main cause of the dupilumab-related conjunctivitis and that targeting SPDEF caused an even further reduction in MUC5B, a mucin with an important role in airway homeostasis and protection from infection [21].

We leave the reader with what we consider the current open research and clinical questions regarding the mucin role in the pharmacotherapy of asthma:

1. What are the role and clinical relevance of the new and upcoming biologics, PDE4 antagonist and prostaglandin D2 receptor antagonists, in the regulation of mucin expression and function in severe asthma?
2. Can DNA sequencing and subsequent targeting of abnormal mucin expression in specific patients with asthma constitute the first ever disease modifier?
3. Is there a role for LAMA in mucus production in patients with severe asthma (or mucus hyper-secretive asthma)?
4. Which are the molecular pathways and pathophysiology linking hyper-reactivity, mucus production, and microbiome dysbiosis in patients with bronchiectasis and asthma features and/or fixed airflow obstruction?
5. May physical therapies such as thermoplasty constitute a valid alternative to reverse airway remodeling in patients with severe asthma?
6. What will be the role of engineered mucins in laboratory microbiome studies and in the development of future antibiotic and probiotic compounds?
